# AI-Aided Design of Carbonated Ductile Well Cement for Enhanced Recovery

**DOI:** 10.1021/acsomega.6c02054

**Published:** 2026-06-12

**Authors:** Yinjian Li, Diego Aparicio, Tianyu Wang, Brandon Byers, Fan Shi, Dustin Crandall, Guoxiang Liu, Qingxu Jin

**Affiliations:** † Department of Civil and Environmental Engineering, 3078 Michigan State University, East Lansing, Michigan 48824, United States; ‡ Resilient, Intelligent, Sustainable & Energy-efficient (RISE) Infrastructure Materials Lab, MSU, East Lansing, Michigan 48824, United States; § School of Sustainable Engineering and the Built Environment, 7864 Arizona State University, Tempe, Arizona 85281, United States; ∥ 17213 National Energy Technology Laboratory, Pittsburgh, Pennsylvania 15236, United States

## Abstract

The long-term integrity of wellbores in enhanced oil recovery and enhanced gas recovery is critically dependent on the mechanical resilience of the cement sheath against tensile subsurface loads. This study developed a series of mix designs for ductile well cements using American Petroleum Institute Class H cement, silica flour, and fly ash and reinforced with polyethylene fibers to address the inherent brittleness of traditional oil well cements. The mechanical behavior and microstructural evolution of the composites were evaluated under normal/ambient and carbonation (10% CO_2_) curing conditions using uniaxial tensile testing, thermogravimetric analysis, and X-ray diffraction. Results indicated that PE fiber reinforcement transformed the failure mechanism from brittle fracture to ductile strain hardening, achieving a peak tensile ductility of 6.3% in the ternary blend after 7 days of normal curing. Carbonation curing accelerated hydration kinetics and matrix densification through extensive calcite precipitation, resulting in a CO_2_ uptake of 17.4 wt % by 28 days. While matrix densification limited the strain capacity to approximately 4%, carbonation curing stabilized the strain-hardening behavior across all test ages, effectively preventing the late-age embrittlement observed in air-cured specimens. An interpretable supervised machine learning framework was further employed to establish quantitative links between hydration and carbonation-driven microstructural evolution and the resulting strength and ductility. These findings demonstrate that fiber-reinforced Class H composites offer huge potential to improve zonal isolation and circular economy viability in CO_2_-rich subsurface environments.

## Graphical abstract

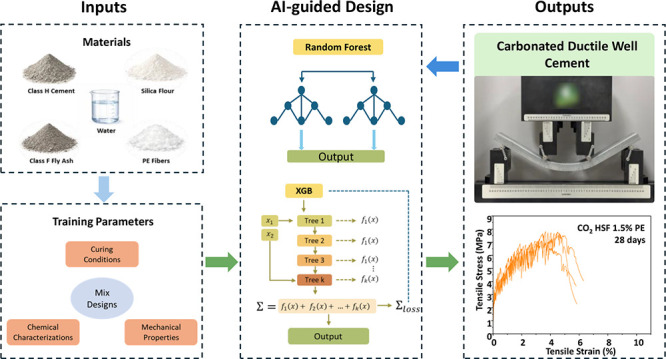

## 1 Introduction

The long-term viability of subsurface activities like enhanced oil recovery (EOR) and enhanced gas recovery (EGR) operations depends critically on wellbore integrity over time scales extending from decades during active injection to centuries during postclosure monitoring.^1,2^ Under downhole conditions associated with CO_2_-injection EOR/EGR, cement degradation driven by carbonation and bicarbonation can contribute to microcracking, increased permeability, and reduced well integrity during injection and production operations.^3^ The U.S. Department of Energy has identified that wellbore integrity represents the greatest challenge to ensuring safe and reliable injection operations and long-term containment in subsurface complexes.^4^ Field surveys indicate that legacy wells, particularly those drilled before 1950 when no industry plugging standards existed, pose elevated risks due to casing corrosion and cement degradation.^5,6^

Injected fluids and formation brines induce corrosion to metallic tubulars and cement in the wellbore, while mechanical integrity loss occurs due to cyclic and thermal loading throughout the well life.^7^ Intermittent injection operations, common in onshore and offshore settings, subject wellbores to pressure cycling as injection pressure varies between high-flow and buffer periods.^8^ This cyclic loading, combined with thermal stresses from operations at temperatures often exceeding 105 °C and pressures up to 70 MPa,^3^ creates conditions where traditional Portland cement systems experience debonding at casing-cement interfaces and microannulus formation.^9,10^ Temperature changes can break the cement-casing bond, causing a microannulus that serves as a preferential pathway for fluid migration.^10^ Field observations from the Scurry Area Canyon Reef Operators Committee (SACROC) unit, a longest and conventional CO_2_-EOR operation, revealed corrosion along the casing-cement interface after over 30 years of CO_2_ exposure,^11^ demonstrating the long-term consequences of these coupled chemical-mechanical degradation processes.

The circular economy model, emphasizing reduction, reuse, and recycling of materials, offers a framework for extending the life cycle of subsurface resources and infrastructure.^12,13^ Including extensive repurposing abandoned/legacy wells for energy storage, geothermal energy production, or fluid disposal represents a key application of circular economy principles in petroleum operations. However, realizing this potential requires cement systems that maintain integrity through multiple use cycles and extended service lives. Traditional well abandonment practices often fail to account for potential future reuse, resulting in suboptimal material selection that limits the adaptability of existing wellbore infrastructure.^14^

Despite decades of research on durable cement formulations, a critical gap persists in understanding cement performance under complex stress states encountered in EOR/EGR wells. Most studies emphasize compressive strength, while properties like tensile strength, flexural strength, elastic modulus, and bond strength are often neglected.^3^ This research bias is problematic because the fundamental failure mechanism of wellbore cements in practice is not compressive crushing but rather tensile cracking and brittle fracture under bending moments induced by casing deformation, thermal expansion mismatch, and formation movement, particular with various compositions.^15,16^ The inherent brittleness of Portland-based oil well cements render them susceptible to catastrophic failure under cyclic loading, where progressive cracking leads to sudden loss of structural integrity.^17,18^ Ductility, defined as the ability to undergo plastic deformation and redistribute stresses before failure, is therefore paramount for cement systems subjected to the repetitive thermal and pressure cycles characteristic of subsurface energy applications and operations.^19,20^ However, conventional Class G and Class H oil well cements exhibit poor ductility and low fracture toughness, resulting in brittle failure with minimal energy absorption.^21,22^ The predominance of compressive strength testing in literature reflects laboratory convenience rather than field relevance, leaving practitioners without adequate data to predict cement performance under the multiaxial stress states and cyclic loading conditions that govern actual wellbore failures.

Fiber reinforcement presents a promising avenue to address the brittleness problem by fundamentally altering the failure mechanism from brittle to pseudoductile behavior.^23–27^ Research has demonstrated that various fiber types can significantly enhance the mechanical properties and energy absorption capacity of oil well cements. Wollastonite fiber additions at 15% content increased compressive strength by 34.64%, flexural strength by 98.79%, and tensile strength by 46.46% in Class G cement cured at 90 °C.^21^ Hybrid wollastonite-carbon fiber systems showed improved performance, with the fibers randomly distributed in the cement paste and providing reinforcement through bridging and pull-out mechanisms.^28^ Cellulose nanofibers increased flexural strength by 20.7% at only 0.04 wt % addition,^29^ while alumina nanofibers enhanced both mechanical and rheological stability,^18^ both using Class H cement. Recent work on basalt fiber reinforcement has shown promise, with optimized dispersion techniques improving compressive strength and reducing brittleness.^22,30^ Surface modification of basalt fibers with nano-SiO_2_ further enhanced interfacial bonding with the cement matrix of oil well cement and the flexural strength by 37.5%.^31^ Aluminosilicate fibers combined with silane coupling agents (KH560) increased flexural strength by 22.79% at 80 °C through synergistic modification effects.^32^ Polyethylene (PE) fibers can improve corrosion resistance in engineered cementitious composites by inducing fiber-bridging tensile ductility and tightly controlled multiple microcracks that limit crack width, retain rust, and reduce steel mass loss and corrosion propagation.^33^ These improvements stem from the bridging effect of fibers across microcracks, enhanced interfacial bonding, and the ability of the matrix to redistribute stresses and arrest crack propagation, thereby imparting ductility to an otherwise brittle material.^21,29,32^ Importantly, fiber reinforcement has been shown to stabilize cement properties under elevated temperatures relevant to geothermal and deep formation applications.^19^

Studies^34,35^ have demonstrated that incorporating silica flour and fly ash enhances the thermal stability and microstructural integrity of American Petroleum Institute (API) Class H cement under high-temperature conditions. However, previous work has not sufficiently characterized the crack behaviors that are essential for understanding long-term performance under the stress variations characteristic of wellbore operations. Critically, CO_2_ exposure is unavoidable in many EOR/EGR and CO_2_-rich gas wells, where injected or produced CO_2_ rapidly reacts with early age cement to alter hydration, porosity, and long-term sealing performance.^3,36^ Building upon these findings, the present research developed several workable fiber reinforced formulations using API Class H cement, silica flour, fly ash, and polyethylene (PE) fibers to evaluate their feasibility as ductile sealing materials for enhanced oil and gas recovery (EOR/EGR) wellbores. Despite these advances, quantitatively linking hydration- and carbonation-driven microstructural evolution to macroscopic strength and ductility remains challenging, owing to the coupled effects of mixture design, curing conditions, and evolving cement chemistry. Interpretable supervised machine learning offers a data-driven approach to capture these complex, nonlinear relationships and to relate experimentally measurable microstructural descriptors to mechanical performance in a unified framework.^37–40^

This study introduces Class H cement-based ductile well cements (DWCs) reinforced by PE fibers and investigates the compressive behavior, tensile behavior, and crack propagation characteristics under different mix designs and carbon curing conditions. Specifically, we examine: (1) how PE fiber reinforcement affects compressive strength, tensile strength, elastic modulus, and strain capacity; (2) the effects of carbon curing on mechanical performance; and (3) the measurable mechanical advantage of pozzolanic material over only cement, when combined with fibers. In addition, an interpretable supervised machine learning framework is employed to quantitatively link hydration- and carbonation-driven microstructural evolution, characterized by thermogravimetric and XRD, with strength and ductility. By combining Class H cement with supplementary cementitious materials (e.g., fly ash and silica flour) and PE fibers, this work proposes a new ductile well cement formulation that exhibits enhanced ductility in a lab environment that could be potentially used for EOR/EGR applications.

## 2 Experiments and Methods

### 2.1 Materials

#### 2.1.1 Raw Materials

Chemical compositions of the ductile well cements matrix using API Class H cement (API Spec 10A), fly ash (Class F, ASTM C618) and silica flour (200 mesh size) used in the study are listed in Table 1. Deionized (DI) water supplied by Thermo Scientific Barnstead was utilized for the preparation of all solid constituents and admixtures. An ultrahigh molecular weight, high modulus polyethylene (PE) fiber was incorporated into the mixture. The physical and mechanical properties of this hydrophobic fiber, as provided by the manufacturer, are summarized in Table 2. The particle size distributions of ductile well cements constituents were characterized using a Malvern Mastersizer 3000E equipped with an Aero M dry dispersion unit, operated at a feed rate of 50% and a vacuum pressure of 2 bar. Laser obscuration was maintained within the range of 0.1–15%, and measurements were conducted under the general-purpose analysis mode. The particle size distributions expressed in terms of cumulative volume, presented in Figure 1.

**Table 1:** Chemical Composition of Ductile Well Cement Constituents (Provided by Manufacturers)

**Material**	**SiO**_**2**_ (%)	**Al**_**2**_ **O**_**3**_ (%)	**Fe**_**2**_ **O**_**3**_ (%)	**CaO** (%)	**MgO** (%)	**Others**
H cement	21.6	3.2	4.5	63.6	2.7	4.4
fly ash-F	49.1	21.9	15.8	4.0	1.2	8.0
silica flour	>95–99.9	0	0	0	0	<5

**Table 2:** Physical and Mechanical Properties of PE Fibers

	Diameter (μm)	Length (mm)	Elastic modulus (GPa)	Tensile strength (GPa)	Elongation (%)	Density (g cm^–3^)
PE	18	13	117	2.9	<4.0	0.97

**Figure 1:**
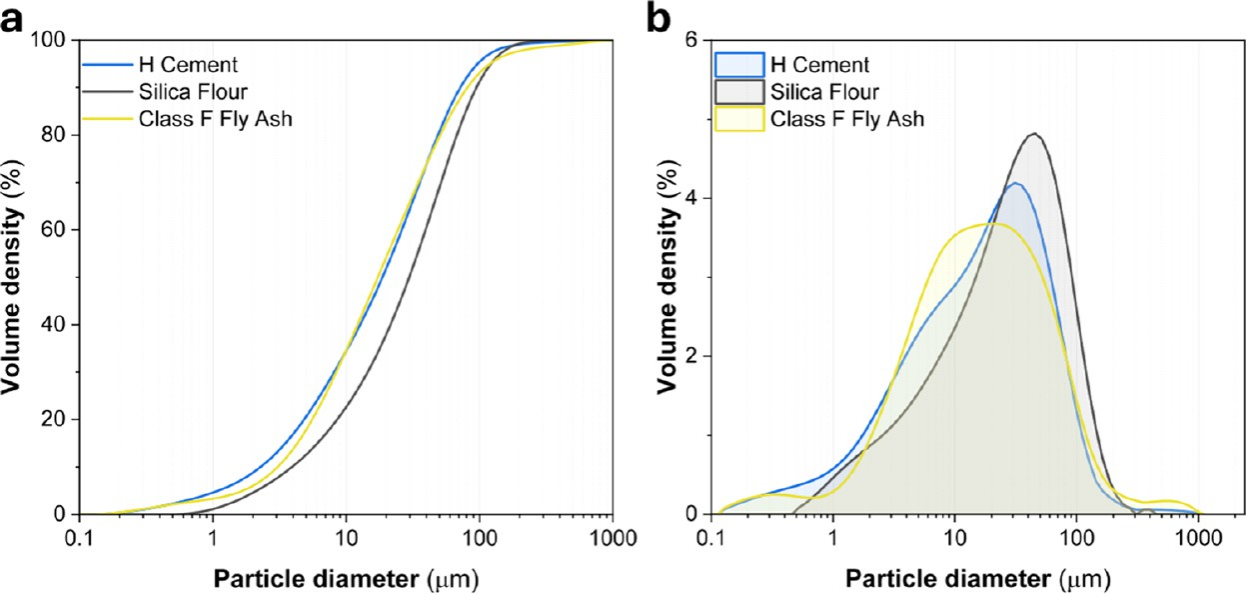
Cumulative (a) and differential (b) particle size distributions of the raw solid materials, including H cement, silica flour, and class F fly ash.

#### 2.1.2 Mix Designs and Sample Preparation

Three sets of DWC mixtures were designed and prepared, as outlined in Table 3. The conventional Class H cement usually has a water to cement ratio of 0.38, but in this study, the water binder ratio for the paste group was set to 0.30 deliberately to provide enough viscosity to support PE-fiber dispersion and composite stability for ductile behavior of the well cement. For the other mix design, 20% of Class H cement was replaced by fly ash to increase workability and prevent bleeding. Silica flour was incorporated to enhance matrix strength and thermal stability, particularly under high-temperature conditions relevant to wellbore applications. As a control (H-1), the pure class H-cement paste with a water binder ratio of 0.30 and 1% PE fiber was used. HSF-1 is the second set of the DWC with class-H cement, fly ash and silica flour. The first two sets are prepared to compare the difference of the combined effect of silica and fly ash while holding the same amount (1%) of fiber. The third set (HSF-1.5) has the same ratio of solids but with 1.5% fiber incorporation, the comparison between set 2 and 3 is to check the effect of fiber volume.

**Table 3:** Mixture Designs for Ductile Well Cements (DWCs)

**Mix ID**	**H cement (**kg/m^3^ **)**	**Fly ash (**kg/m^3^ **)**	**Silica flour (**kg/m^3^ **)**	**Water (**kg/m^3^ **)**	**Fiber vol (**Vol. %**)**
H-1	1924	0	0	577	1.0
HSF-1	1131	283	424	551	1.0
HSF-1.5	1125	281	422	548	1.5

Mixtures were prepared by dry mixing for 2 min. Deionized water was added to the solid constituents for a total mixing time of another 2 min at the same previous speed. PE fibers were carefully added to ensure homogeneous fiber distribution. After adding fibers, the composite was mixed for an extra 4 min to form homogeneous mixtures. Lastly, all fresh mixtures were poured into the molds and after 24 h in an atmospheric condition were demolded. For each subsequent test, three specimens were prepared to ensure consistency.

#### 2.1.3 Sample Curing Conditions

After demolding at 24 h, specimens were subjected to two different curing methods, both at 23 ± 2 °C under laboratory conditions (ambient relative humidity of 50 ± 5%) for 3, 7, and 28 days. The first condition has one set of samples individually sealed in plastic wrap to prevent moisture loss and carbonation, representing a hydration-controlled condition. To evaluate the effect of elevated CO_2_ concentrations on the performance of the bendable cement slurries, the second set was cured under identical temperature and humidity conditions in an Air-Jacketed CO_2_ Incubator (VWR International), where a CO_2_-rich atmosphere was maintained at 10%, providing a controlled carbonation environment.

### 2.2 Chemical Characterization

#### 2.2.1 Thermogravimetric Analysis (TGA)

Thermogravimetric (TGA) and derivative thermogravimetric (DTG) analyses were conducted to identify and measure hydration products evolution of the cement slurries, focusing primarily on the CO_2_ uptake of the carbonated samples. Finely ground powders were extracted from the specimen gage section for analysis. To minimize carbonation prior to testing, hydration was stopped using solvent exchange with isopropanol followed by diethyl ether. After filtering out the diethyl ether with the funnel, the sample was placed in an oven at 40 °C for 15 min to evaporate any remaining alcohol. A mass of ∼30 mg per sample was placed into platinum crucibles, as recommended by ASTM C1872,^60^ and loaded into a thermogravimetric analyzer (TA Instruments TGA 550). The loaded crucibles were predried at an isothermal state of 40 °C for 20 min. Then, the heating process was carried out from 40 to 1000 °C, with a heating rate of 10 °C/min, under an N_2_ atmosphere, with a flow rate of 25 mL/min.

CO_2_ uptake was quantified from the mass loss associated with carbonate decomposition during TGA. The mass loss between 600 and 750 °C, Δ*m*_600–750_, was attributed to the release of CO_2_ from CC̅ decomposition. Therefore, the corresponding mass of CO_2_ released, *m*_CO_2__, was taken directly as the measured mass loss in this temperature interval, as expressed in eq 1.

$$m_{{\mathrm{CO}}_{2}}=\Delta m_{600-750}$$ 1

The CO_2_ uptake was then calculated according to eq 2, in which the net CO_2_ released from the carbonated sample was obtained by subtracting the corresponding contribution from the noncarbonated reference and then normalized by the mass of paste

$${\mathrm{CO}}_{2}\mathrm{uptake}(\mathrm{wt}.\%\mathrm{of}\mathrm{paste})=\frac{m_{{\mathrm{CO}}_{2},\mathrm{carbonated}}-m_{{\mathrm{CO}}_{2},\mathrm{non}-\mathrm{carbonated}}}{m_{\mathrm{paste},750}(1+w/b)}\times 100$$ 2

where *m*_CO_2_,carbonated_ is the mass of CO_2_ released from the carbonated paste sample, *m*_CO_2_,non‑carbonated_ is the mass of CO_2_ released from the corresponding noncarbonated reference sample, *m*_paste,750_ is the mass of the paste sample at 750 °C, and *w*/*b* corresponds to the water-to-binder ratio of the slurry, following directions used in.^41^

#### 2.2.2 Isothermal Calorimetry

Isothermal calorimetry was performed to quantify the hydration kinetics of the cement slurries. By measuring heat flow and cumulative heat, the analysis focused on the induction period, acceleration peak(s), and late-age hydration behavior of supplementary cementitious materials (SCM) additions. Cumulative heat at fixed ages (12, 24, and 72 h) was used as a reference for the degree of hydration, and, together with TGA-derived CH consumption, provided a quantitative assessment of the pozzolanic contribution of the SCMs. All calorimetry tests were conducted on sealed vials maintained at 23 °C, and two replicates were performed in accordance with ASTM C1679 ^42^ and ASTM C1702.^43^

#### 2.2.3 X-ray Diffraction (XRD)

X-ray diffraction (XRD) characterization was included to identify the crystalline phases developed in the Class H bendable slurries over time. Approximately one gram of the hydrated slurries was finely ground by hand to obtain a uniform fine powder (<75 μm). The samples were subjected to a hydration stoppage treatment using the same approach detailed in Section 2.2.1. Afterward, the powders were loaded into XRD sample holders and analyzed using a Rigaku SmartLab diffractometer equipped with Cu Kα radiation (λ = 1.54 Å, 40 kV, 44 mA). Data were collected over a 2θ range of 5–50°, employing a one-dimensional detector with a scan rate of 3° min^–1^.

### 2.3 Mechanical Characterization

#### 2.3.1 Compressive Strength Test

Compressive strength was evaluated using 50 × 50 × 50 mm cubic specimens (width × length × height) tested after 3, 7, and 28 days of curing. For each mixture, three cubes with PE fibers were prepared to examine the influence of fiber incorporation on compressive strength. The tests were carried out using a Forney automatic compression testing machine, under a constant loading rate of 0.542 MPa s^–1^ in accordance with ASTM C109/C109M-20.^44^ The samples were cast in metal molds, and the flat faces that were cast against the mold walls were used as loading faces.

#### 2.3.2 Uniaxial Tensile Test

Uniaxial tensile tests were conducted using an electromechanical load frame equipped with a 50 kN load cell. Dogbone-shaped specimens were employed to promote crack formation in the central section under tensile loading. The dimensions of the specimen are shown in Figure 2a. Each specimen was mounted in specially designed clamps, as illustrated in Figure 2b. The tests were performed under displacement control at a loading rate of 0.50 mm min^–1^, following the recommendations of Japan Society of Engineers (JSCE),^45^ to simulate quasi-static loading conditions. Two linear variable differential transformers (LVDTs), each with a displacement capacity of 15 mm, were positioned adjacent to the specimen’s gage section to measure local deformation. The total strain was determined by averaging the displacements recorded by both LVDTs and dividing by the initial gage length. Stress–strain curves were generated from the measured load and displacement data, where stress (MPa) was obtained from the load cell and strain (%) from the LVDTs.

**Figure 2:**
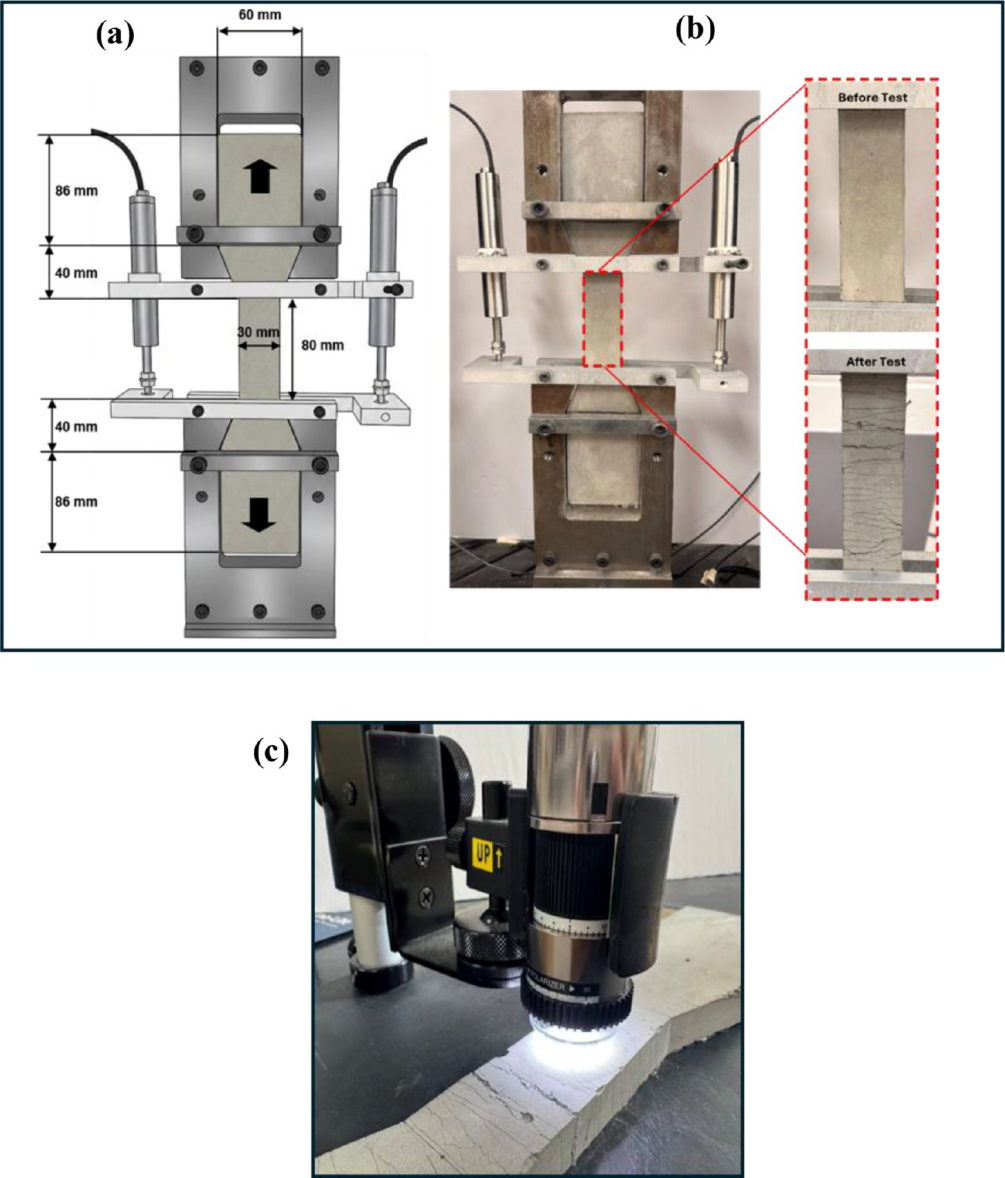
(a) Dimensions of dog-bone-shaped specimen for uniaxial tension test. The thickness of each dog bone sample is 13 mm. (b) Uniaxial tensile test setup equipped with linear variable differential transformers (LVDTs) for deformation measurement. The inset shows the specimen before and after testing, highlighting the formation of multiple microcracks. (c) Optical microscopy setup used for surface crack pattern characterization on the tested specimens.

#### 2.3.3 Residual Crack Pattern Characterization

Crack pattern characterization was performed by quantifying crack width and crack number in the unloaded state. Measurements were conducted using a Dino-Lite Edge Plus digital microscope with a resolution of 9 μm per pixel, as shown in Figure 2c. After tensile testing, specimens were removed from the setup and examined along a predrawn centerline in the longitudinal direction of the dogbone, following the procedure described by Leon-Miquel et al.^27^ Results were presented as histograms showing the number of cracks per 20 μm width interval.

### 2.4 Machine-Learning-Based Property Analysis

To establish quantitative links between mixture design, microstructural characteristics, and mechanical performance of strain-hardening oil well cement composites, a supervised machine learning framework was adopted, as shown in Figure 3. The analysis focused on three mechanical responses: compressive strength, tensile strength, and tensile strain capacity. Input variables were selected to span formulation, curing, and microstructural scales, including binder composition, fiber volume fraction, curing age and condition, as well as descriptors derived from TGA and XRD. The latter captured key aspects of hydration and carbonation through characteristic mass-loss intervals and normalized diffraction peak intensities associated with major phases.

**Figure 3:**
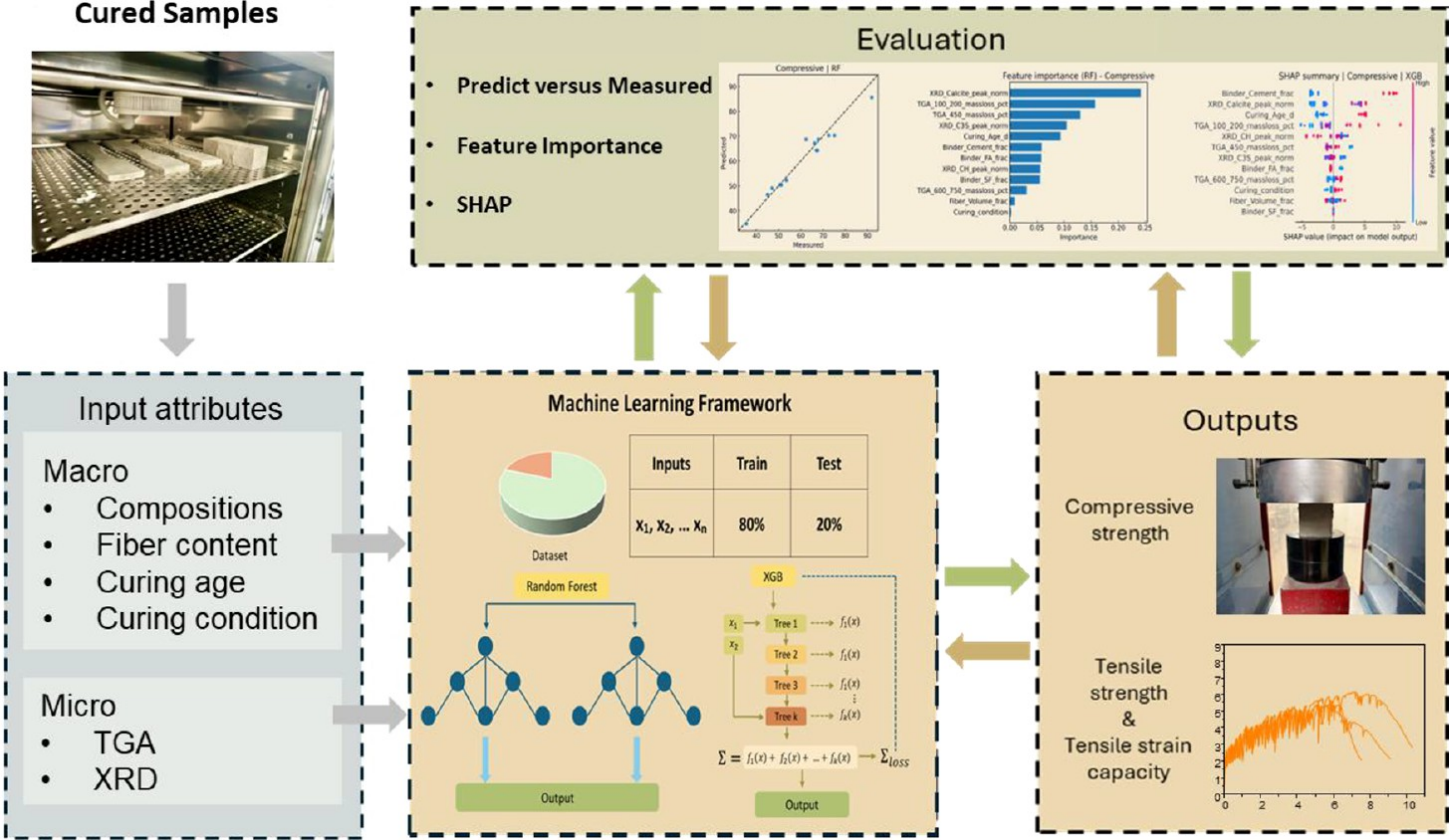
Schematic illustration of the conducted machine learning framework.

Random Forest (RF) and Extreme Gradient Boosting (XGBoost, XGB) models were applied due to their suitability for limited experimental data sets and their ability to represent nonlinear relationships without presupposed functional forms. The data set was randomly divided into training (80%) and testing (20%) subsets, and identical feature sets were used for both models to enable direct comparison. Model performance was evaluated using the coefficient of determination (*R* ^2^), mean absolute error (MAE), and root-mean-square error (RMSE), which collectively quantify goodness-of-fit, average prediction deviation, and sensitivity to larger prediction errors, respectively. In addition, predicted-versus-measured relationships and residual analyses were used to assess accuracy and potential systematic bias.

To interpret model behavior, feature importance metrics were extracted from both RF and XGB models. Furthermore, SHapley Additive exPlanations (SHAP) were applied to the XGB models to quantify both the magnitude and direction of individual feature contributions.^46,47^ This integrated modeling and interpretation strategy enables the machine learning results to be directly related to experimentally observed hydration chemistry, microstructural evolution, and mechanical behavior, rather than serving solely as predictive tools.

## 3 Results and Discussion

### 3.1 Chemical Characterization

#### 3.1.1 Isothermal Calorimetry Results

Figure 4 shows the hydration kinetics of the pastes during 7 days of hydration measured by isothermal calorimetry, where the heat flow curves highlight a clear difference in early age hydration kinetics between the H paste and the ternary HSF blend. The Class H paste exhibits a pronounced main hydration peak associated with the accelerated dissolution of C_3_S[Fn fn1] and the formation of C–S–H and portlandite (Ca­(OH)_2_ or CH), whereas the HSF paste shows a reduced peak magnitude and slower evolution of the early hydration stages. This behavior is attributed to the combined effect of clinker dilution and the slower reactivity of the SCMs used. In the HSF system, since a fraction of the cement is replaced by fly ash and silica flour, the fly ash is mainly composed of a glassy structure and dissolves slowly in the alkaline pore solution, contributing minimally to the main hydration peak and acting primarily as a filler during the first days.^48^ At the same time, the silica flour, while not inert, is present as relatively coarse crystalline SiO_2_ particles with low specific surface area, so its dissolution and participation in early pozzolanic reactions is also limited. Similar trends have been reported for blended systems with high replacement levels of pozzolans or limestone, where coarser particles do not significantly participate in early hydration and the early heat flow is governed by a filler/nucleation effect, while the cumulative heat per mass of binder decreases due to dilution of the clinker phase.^49^

**Figure 4:**
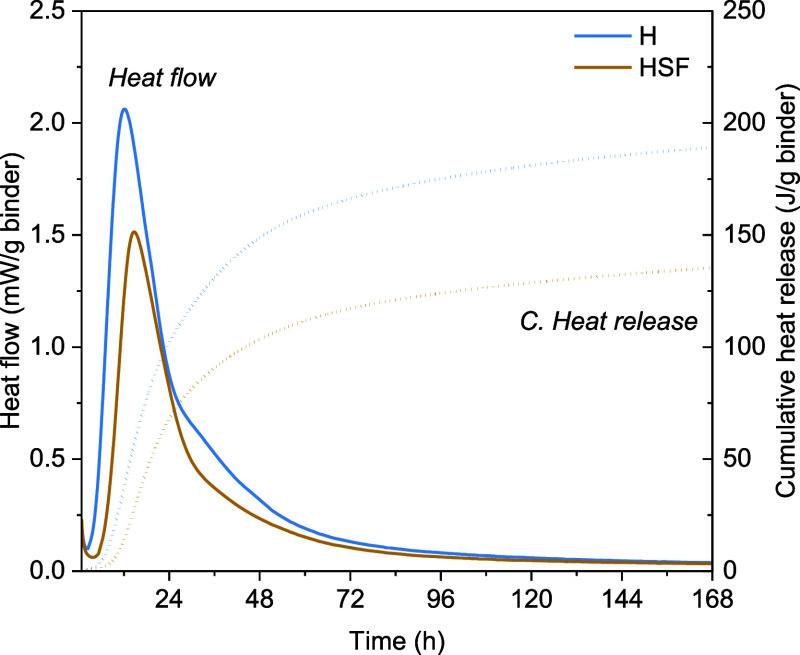
Heat flow curves of H and HSF pastes. Two replicate tests were performed for each sample type; the resulting curves are identical.

Because the heat flow is normalized per gram of paste, it is reasonable that the HSF mixture shows lower hydration kinetics, where a portion of the highly reactive clinker is replaced by more slowly reactive SCMs. However, this slower early reaction is not necessarily detrimental. The SCMs provide a filler effect and additional nucleation sites for hydration products, where the reduced cement content leaves more free water and pore space available for continued hydration. As a result, the HSF system can still develop a dense microstructure and substantial hydration products at later ages.

#### 3.1.2 Thermogravimetric Results

Thermogravimetric analysis with differential thermogravimetry (TGA/DTG) was used to identify hydration and carbonation products in the pastes at 3, 7, and 28 days. The DTG curves (Figure 5) reveal three major mass-loss regions corresponding to (i) dehydration of C–S–H gel and ettringite between 100–200 °C; (ii) dehydroxylation of portlandite (CH) at around 450 °C; and (iii) decomposition of carbonate phases between 600–750 °C (primarily calcite, CaCO_3_ or CC̅).^41^ Both H and HSF samples exhibit these characteristic peaks, but their intensities vary with mix composition, age, and curing conditions. By 3 days (Figure 5a), the normal-cured H paste shows a prominent CH peak and only a minor carbonate decomposition due to minimal exposure to CO_2_, whereas the carbonated H paste has a reduced CH peak and a noticeable weight-loss peak in the 600–750 °C range from CO_2_ uptake (CC̅ formation). A similar behavior is observed for the HSF paste, with the difference that the weight losses associated with C–S–H and CH are lower compared to the H paste, due to the lower cement content and slower pozzolanic reactions of the SCMs. At higher temperatures (750–900 °C), both pastes show minor additional mass losses, which are attributed to the decomposition of C–S–H into wollastonite; this peak was intensified in the H paste, consistent with its higher degree of cement hydration.

**Figure 5:**
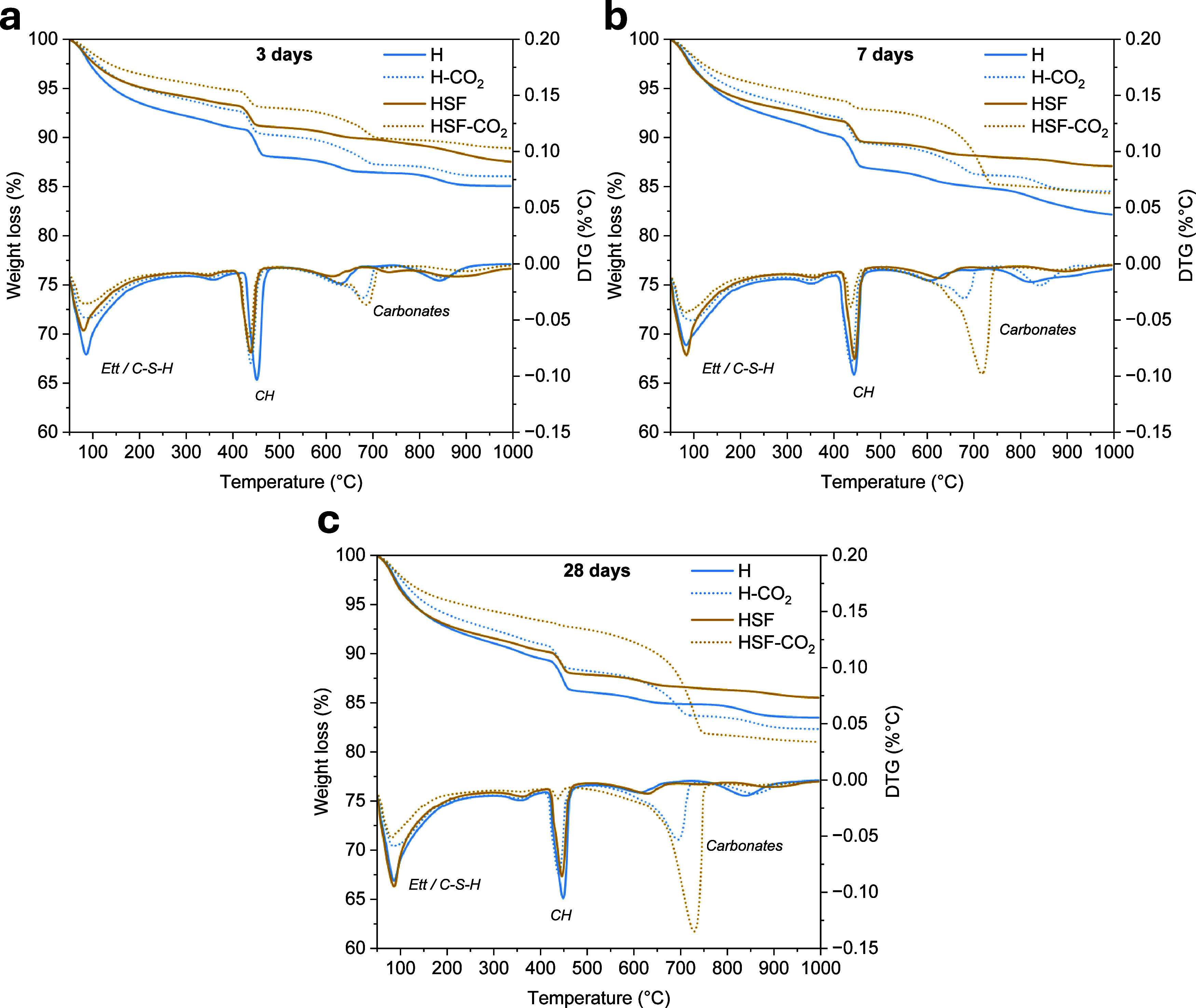
Weight loss and derivative thermogravimetry (DTG) curves for cement pastes after 3 (a), 7 (b), and 28 (c) days of curing. Solid lines correspond to the samples cured under normal conditions, while dashed lines correspond to samples cured under elevated (10%) CO_2_.

After 7 days (Figure 5b) the normal-cured H paste remained relatively unchanged, with only minor reductions in the CH peak attributed to continued CH consumption during C–S–H formation. In contrast, the carbonated samples exhibited a different behavior. Notably, both H–CO_2_ and HSF-CO_2_ pastes show reduced C–S–H mass loss (100–200 °C region) compared to their normal-cured counterparts, indicating that carbonation competes with hydration by preferentially consuming CH before it can participate in pozzolanic reactions to form additional C–S–H. This competitive mechanism is particularly pronounced in the HSF-CO_2_ system, which exhibited significantly higher carbonate mass loss than H–CO_2_. The enhanced carbonation in the HSF blend can be attributed to several factors: (i) cement dilution reduces the overall CH buffering capacity, making available CH more susceptible to carbonation; (ii) slower pozzolanic reactions of fly ash mean that CH remains available for longer periods, extending the window for CO_2_ uptake; and (iii) the delayed hydration results in a more porous microstructure during early ages, facilitating deeper CO_2_ diffusion into the paste.^50,51^ While fly ash has been reported to increase carbonation susceptibility in blended systems, the silica flour acts primarily as a physical diluent, contributing to the reduced CH content but not directly participating in carbonation reactions.^52,53^

By 28 days (Figure 5c), the normal-cured H paste exhibited well-defined C–S–H and CH peaks, indicative of mature hydration. The carbonated pastes, however, continued to show suppressed C–S–H formation relative to ambient conditions, with the reduction being more pronounced in HSF-CO_2_. While the H–CO_2_ paste showed moderate carbonation with residual CH still present, the HSF-CO_2_ sample displayed near-complete disappearance of the CH peak accompanied by a substantial increase in carbonate decomposition (600–750 °C). The near-exhaustive CH depletion in the ternary blend allowed for extensive CC̅ precipitation but simultaneously limited the potential for continued C–S–H formation through pozzolanic reactions. This explains why, despite ongoing hydration from 7 to 28 days, the C–S–H content in carbonated samples remains lower than in normal-cured samples, where the available CH is consumed by carbonation rather than pozzolanic reactions.

The CO_2_ uptake derived from the carbonate decomposition region (600–750 °C) was quantified using eqs 1 and 2, accounting for baseline carbonate content in noncarbonated reference samples, and is summarized in Figure 6. At 3 days, both systems show modest initial CO_2_ uptake: 2.4 wt % for H–CO_2_ and 2.8 wt % for HSF-CO_2_, reflecting early carbonate formation. The divergence becomes more evident by 7 days: while H–CO_2_ shows essentially no further uptake (2.3 wt %), indicating carbonation has stalled due to surface layer densification,^52^ the HSF-CO_2_ paste exhibits a sharp increase to 11.0 wt %, a 293% increase from 3 days. This suggests that the HSF microstructure remains sufficiently open to allow deep CO_2_ penetration during this period when CH is still abundant but pozzolanic reactions have not yet densified the pore structure. By 28 days, the H–CO_2_ paste shows gradual increase to 6.2 wt % (a 170% increase from 7 days), while the HSF-CO_2_ system reaches 17.4 wt % (a 58% increase from 7 days), nearly triple the uptake of H–CO_2_ at equivalent age. This difference can be partly explained by the mixture proportions. Compared with H, the HSF paste contains 41% less cement, which lowers the theoretical CH reserve available for carbonation. Nevertheless, the net CH measured by TGA reflects the combined effects of dilution, hydration, fly ash reaction, and carbonation, and therefore does not scale directly with cement reduction alone. The greater CO_2_ uptake of HSF is thus interpreted as the result of reduced alkaline reserve together with higher carbonation susceptibility of the blended system.

**Figure 6:**
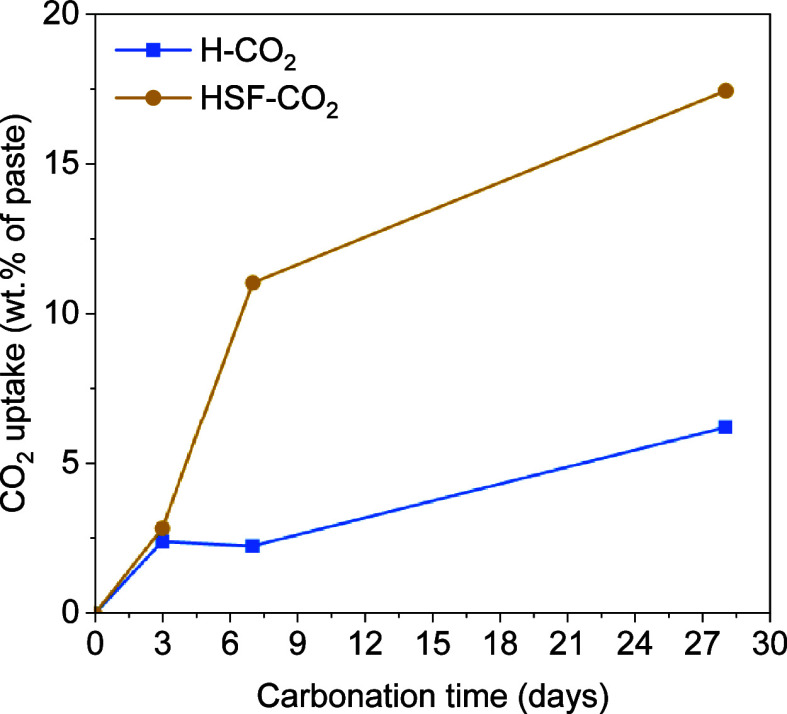
Estimated CO_2_ uptake evolution of H and HSF pastes.

#### 3.1.3 X-ray Diffraction Results

X-ray diffraction (XRD) patterns of H and HSF cement pastes after curing under air and 10% CO_2_ are shown in Figure 7. Both samples exhibited reflections characteristic of hydrated portland cement systems, with dominant peaks corresponding to CH near 2θ ≈ 18°, 34°, and 47° and residual C_3_S in the range 2θ ≈ 32–33°. In the CO_2_-cured specimens the intensities of the CH reflection were lower compared with the air-cured, confirming the partial consumption of CH through early carbonation. In addition, the peak attributable to CC̅) 2θ ≈ 29.4° was more pronounced in the CO_2_-cured samples, which aligns with the increased decarbonation peak observed in the TGA results (Figure 5).

**Figure 7:**
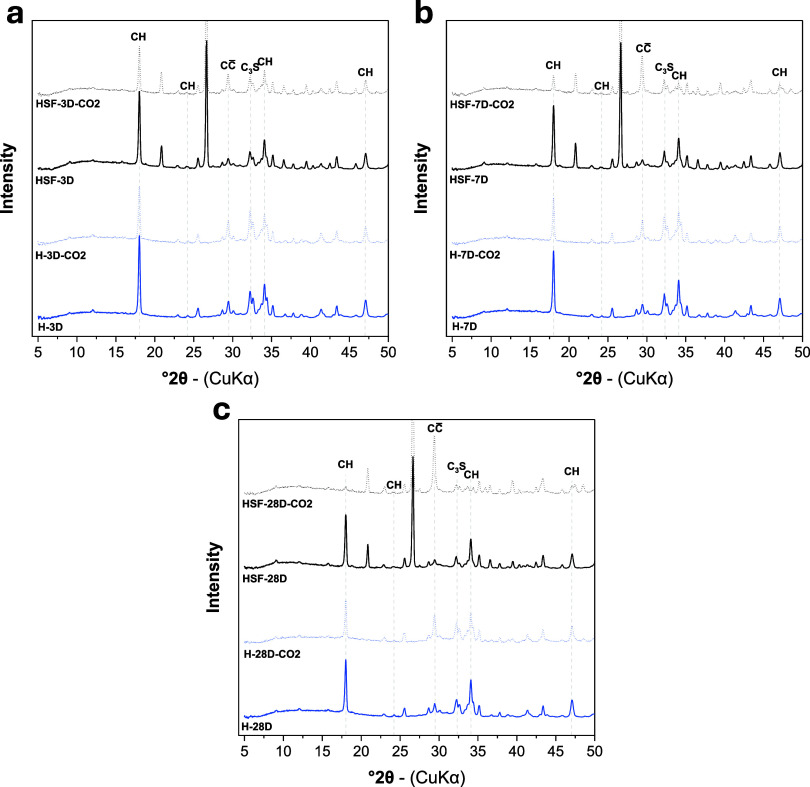
X-ray diffraction (XRD) patterns of H and HSF pastes after 3 (a), 7 (b), and 28 (c) days of curing, highlighting the main crystalline phases **CH** = portlandite, **CC̅** = calcite, and **C**_**3**_ **S**.

At 3 days (Figure 7a) under normal curing, both H and HSF show clear CH peaks, with higher intensity in the H paste, consistent with its higher cement content and faster early hydration. The HSF pattern exhibits similar CH and less pronounced C_3_S reflections, indicating a higher degree of clinker hydration produced by the filler effect of the SCMs. After the 10% CO_2_ exposure, the intensity of the CH reflections decreases in both mixes, while the CC̅ peak becomes more pronounced. This confirms CH depletion and early formation of carbonate phases in agreement with the TGA observations. The reduction in CH is more evident in the HSF sample, reflecting an enhanced carbonation capability in the blended system.

After 7 days (Figure 7b), the normal-cured H paste continues to show strong CH reflections and reduced C_3_S intensity relative to 3 days, indicating ongoing hydration of the silicate phases. The HSF sample, however, shows comparatively weaker CH peaks, pointing to both lower CH generation and partial consumption by the pozzolanic reaction of the SCMs to form C–S–H. Under CO_2_ curing, the contrast between H and HSF becomes more pronounced. In H–CO_2_ paste, CH peaks are substantially reduced but still detectable, with a concurrent increase in CC̅ intensity. In the HSF-CO_2_, CH reflections are nearly depleted, and CC̅ becomes the dominant crystalline phase in the pattern. This behavior is consistent with the higher carbonate mass loss observed in TGA for the HSF-CO_2_ and indicates that the ternary blend experiences a more advanced state of carbonation than the neat H paste at the same age.

At 28 days (Figure 7c), the normal-cured H paste retains strong CH reflections at 2θ ≈ 18° and 34°, while the HSF-CO_2_ sample is dominated by the CC̅ peak at 29.4° and shows an almost complete disappearance of CH reflections. In contrast, the H–CO_2_ sample exhibits only a partial reduction in CH intensity. These results indicate substantially more extensive carbonation in the HSF blend, likely due to its lower initial CH reserve associated with cement dilution. The persistence of C_3_S reflections in the CO_2_-cured samples indicates that unhydrated silicate phases remained detectable throughout the studied period, while carbonation primarily affected CH and promoted CC̅ formation.

Overall, the XRD and TGA results consistently show that exposure to 10% CO_2_ accelerated CH consumption and CC̅ formation, with a much stronger effect in the HSF blend than in the H paste. The blended system therefore exhibited greater carbonation extent over the studied period, which is consistent with its higher measured CO_2_ uptake.

### 3.2 Mechanical Characterization

#### 3.2.1 Compressive Strength

Under normal curing conditions, the H-1 cement paste consistently outperforms the HSF composites, as the pure cement hydration product is most concentrated. As illustrated in Figure 8a, H-1 specimens achieved the highest compressive strength across all ages, peaking at 74 MPa (28 days) compared to 53 and 48 MPa for HSF-1 and HSF-1.5, and this can be attributed to the lower water to cement ratio that actually increased ion concentration for the hydration reaction. The implementation of CO_2_ curing significantly accelerates all stage compressive strength development, particularly for the HSF series. For instance, the HSF-1.5 composite exhibited a substantial increase at 3 days, jumping from ∼31 MPa (normal) to ∼41 MPa (CO_2_), and exceeding 51 and 67 MPa by day 7 and day 28. Similar trend was observed for other sets of samples. This indicates that the carbonation reaction effectively densifies the microstructure and significantly developed at an early age, compensating for the slower pozzolanic reaction of the fly ash.

**Figure 8:**
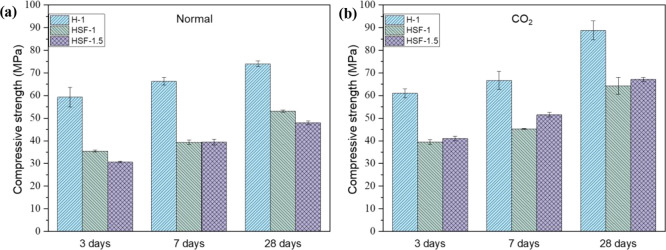
Compressive strength results of H Cement paste with 1% PE fibers, H cement–silica flour–fly ash composite with 1% PE fibers, and H cement–silica flour–fly ash composite with 1.5% PE fibers after 3, 7, and 28 days of normal (a) and CO_2_ curing (b). Error bars represent one standard deviation from the mean of three replicate specimens.

Compared with normal curing, CO_2_ curing also enhances the speed of strength building. This acceleration is most apparent when comparing strength gains between 3 to 7 and 7 to 28 days. Under normal curing (Figure 8a), strength development slowed slower trend (e.g., H-1 gained ∼7 MPa from 3 to 7 days, but only ∼8 MPa from 7 to 28 days). In contrast, CO_2_ curing (Figure 8b) produced a clear second-stage boost: H-1 gained ∼6 MPa early but ∼22 MPa later. HSF series mixes showed the same pattern, with HSF-1.5 nearly doubling its late-age gain under CO_2_ curing (∼16 MPa vs ∼8 MPa). This late-stage acceleration can be attributed to a nucleation seeding effect. The calcium carbonate formed during early CO_2_ curing provides abundant, well-distributed nucleation sites. As noted by Oey et al.,^54^ CC̅ surfaces reduce the energy barrier for C–S–H precipitation, enabling the remaining cement to hydrate more rapidly. Consequently, between 7 and 28 days, C–S–H grows outward from these seeds, promoting faster and more uniform matrix densification than the slower, grain-controlled hydration seen under normal curing.

Critically, the curing environment fundamentally alters the impact of fiber dosage on strength. Under normal curing, for example, increasing the PE fiber content from 1% to 1.5% reduced the 3 day strength from ∼35 MPa to ∼30 MPa. This reduction can be attributed to the hydrophobic nature of PE fibers tending to increase porosity and interconnected voids.^55^ Conversely, under CO_2_ curing (Figure 8b), this trend was reversed, the HSF-1.5 samples not only recovered this deficit but outperformed the HSF-1 formulation. This suggests that the fiber induced porosity acts as a network of diffusion channels during carbonation, providing conduits that facilitate CO_2_ ingress, which is consistent with findings that open pore structures enhance carbonation depth.^56^ These channels could facilitate CO_2_ ingress deeper, allowing the formation of calcium carbonate to densify the matrix bindings.

It is worth noting that the lower water/binder ratio adopted in this study, compared to the API guideline for unreinforced cement slurries, likely contributed to a denser matrix and higher baseline strength. Therefore, the mechanical findings reported here are more appropriately interpreted as conservative rather than compromised by the selected mix design. Moreover, the API-recommended value of 0.38 was originally developed for conventional slurries and does not consider the rheological and dispersion requirements introduced by fiber reinforcement. While this lower water/binder ratio likely influenced the absolute hydration and mechanical response of the system,^57^ all mixtures were evaluated under the same mix-design framework; therefore, the main findings of this study should be interpreted as applying to the developed DWC system rather than to conventional well cement in general.

#### 3.2.2 Tensile Strength and Ductility

The tensile strain–stress properties are shown in Figures 9 and 10. All three mix designs show strain-hardening behaviors both in normal and CO_2_ curing conditions. At early ages, CO_2_-cured samples showed significantly improved strain-hardening behavior with both ductility and ultimate tensile strength. This aligns the observation by Zhang et al.^51^ that CO_2_ in the early ages enhances strength and ductility faster than normal curing. Figure 11 summarizes the evolution of other tensile properties for the three Class H–based composite formulations under both normal and CO_2_-curing conditions including first crack strength, ultimate tensile strength and strain capacities.

**Figure 9:**
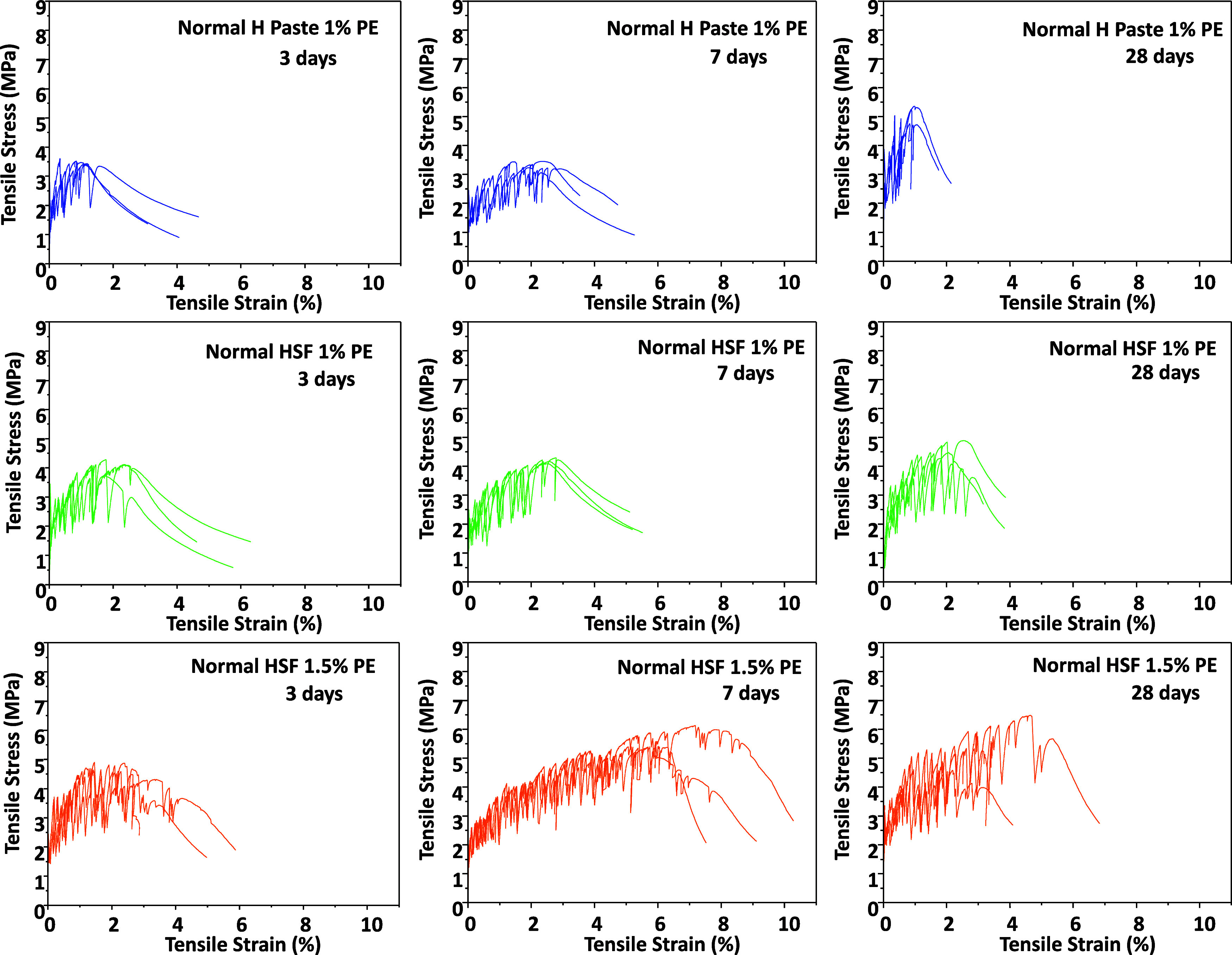
Uniaxial tensile stress–strain curves of H cement–silica flour–fly ash composite reinforced with 1.5% PE fibers after 3 and 7 days of **normal** curing. Three replicates were tested for each age, with each curve color representing an individual test.

**Figure 10:**
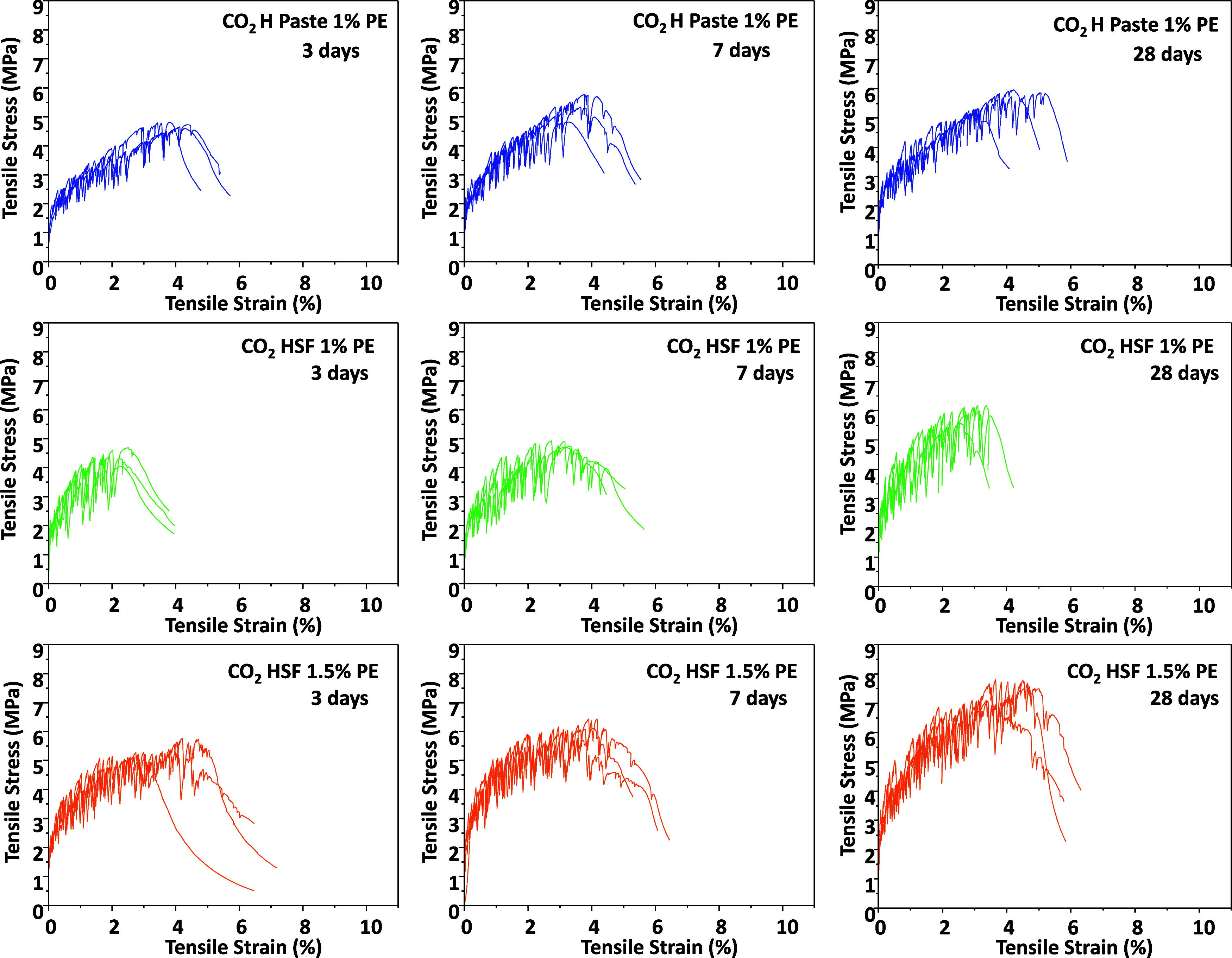
Uniaxial tensile stress–strain curves of H cement–silica flour–fly ash composite reinforced with 1.5% PE fibers after 3, 7, and 28 days of **CO**_**2**_ curing. Three replicates were tested for each age, with each curve color representing an individual test.

**Figure 11:**
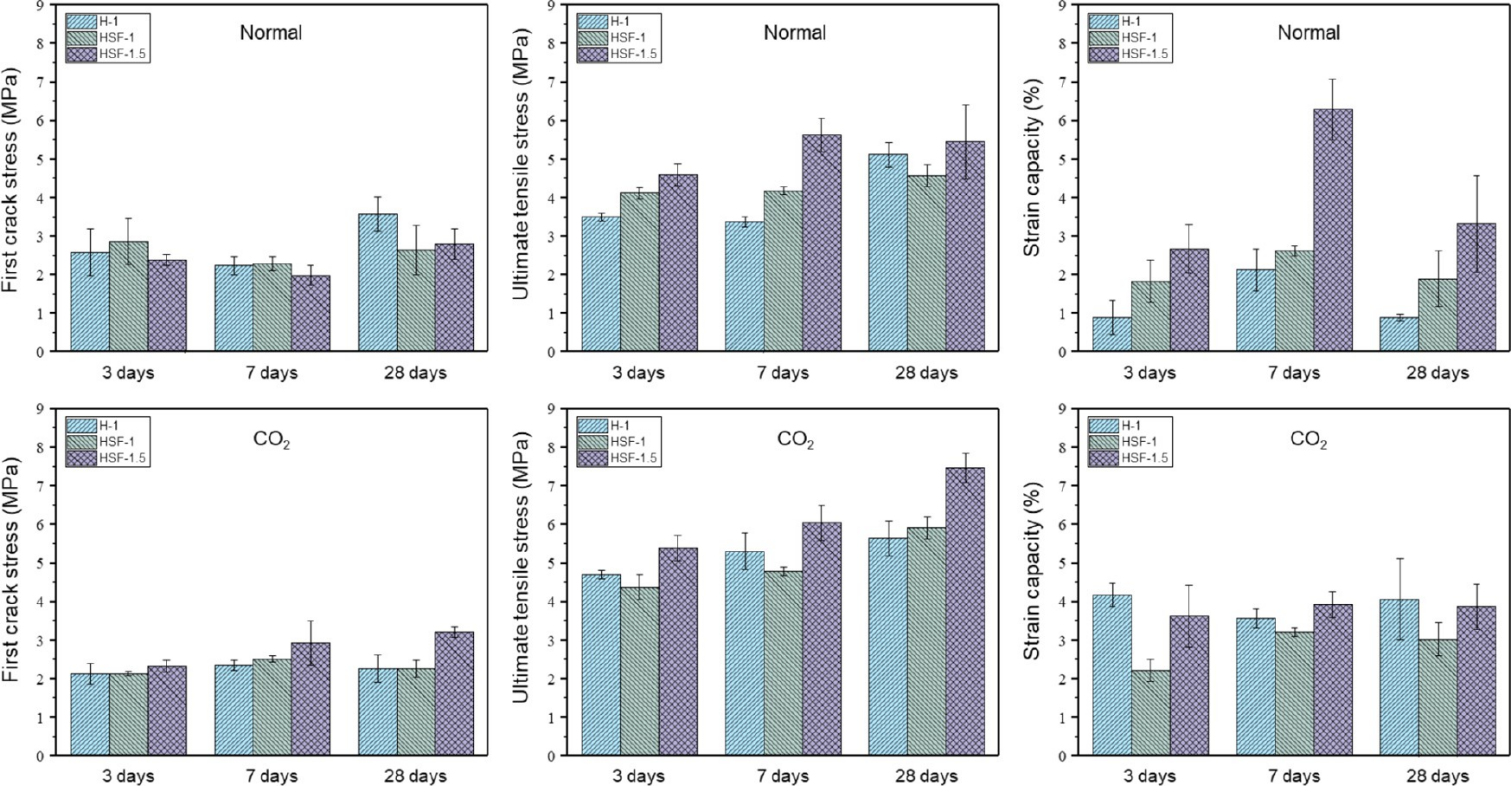
First crack strength, ultimate tensile strength, and strain capacity of each mix design under (upper) normal and (lower) CO_2_ curing conditions. Three replicates were tested for each age.

After 3 days, CO_2_ curing did not significantly increase the first-cracking strength and all three mix designs exhibited first-crack stresses like, or slightly lower than, those under normal curing. This parameter is highly sensitive to local defects that could lead to large experimental scatters. However, CO_2_ curing significantly enhanced the ultimate tensile strength and strain capacity at this age for all three mixtures. The ultimate tensile strength of CO_2_ cured H-Paste reached 4.7 MPa, compared to 3.5 MPa for the normally cured specimen, and the strain capacity increased from 0.9% to 4.18%, suggesting that CO_2_ curing accelerates the densification of the matrix, as also observed in Section 3.1.2. Similar improvements in ultimate strength and ductility were observed for HSF-1 and HSF-1.5.

At 7 days, CO_2_ curing resulted in a slight increase in first-crack strength for all mixtures. For the H-1 samples, CO_2_ curing led to reduced strain capacity compared with normal curing, despite the increase in ultimate tensile strength relative to 3 days. In contrast, HSF-1 exhibited similar ultimate tensile strengths under CO_2_ and normal curing, but with a higher strain capacity under CO_2_ exposure. The HSF-1.5 mixture showed the most pronounced response: normally cured specimens reached their maximum strain capacity of approximately 6.3% at 7 days, while CO_2_ curing increased the ultimate tensile strength by 14.6% but reduced the strain capacity from 6.3% to 3.9%. This reduction reflects earlier crack localization caused by carbonation-induced densification of the matrix and interfacial transition zone, which strengthens the fiber–matrix bond and limits the fiber slip required to sustain large tensile deformation.

At 28 days, CO_2_ curing demonstrates a distinct advantage of ductility stability as well as showing enhanced ultimate tensile strengths. Specifically, the HSF-1.5 mix under CO_2_ curing achieved a peak ultimate tensile stress of approximately 7.5 MPa, markedly outperforming the 5.5 MPa achieved under normal conditions. Furthermore, while the normal-cured HSF-1.5 specimens suffered a sharp decline in strain capacity dropping from a peak of ∼6.3% at 7 days to just 3.3% at 28 days, the CO_2_ cured ones retained a stable strain capacity of roughly 4%.Similarly, in the H-1 samples, CO_2_ curing prevented late-age embrittlement, maintaining a high strain capacity of ∼4.0% even at 28 days compared to the brittle failure (∼0.9%) observed in the normal cured specimens. This phenomenon can be attributed to the fact that CO_2_ curing accelerates early strength gain, but the carbonation reaction competes with hydration by consuming calcium hydroxide and thereby limiting the extent of subsequent hydration.

Overall, in DWC systems, tensile ductility and strength depends on a micromechanical balance between the matrix cracking toughness and the fiber bridging capacity. Under normal curing, ongoing hydration densifies the matrix and generally increases matrix stiffness, strength, and fracture resistance. The superior strain capacity of HSF samples at 7 days is attributed to the fact that this age most closely satisfies the micromechanical conditions required for pseudostrain-hardening, namely a favorable balance between matrix fracture resistance and fiber-bridging behavior. When matrix aging increases the resistance to crack initiation and propagation, while simultaneously strengthening the fiber–matrix interfacial bond, fiber pullout becomes more restricted, and deformation tends to localize into fewer cracks. As a result, the multiple-cracking behavior becomes less stable, fiber bridging becomes less effective in sustaining distributed cracking, and the composite exhibits reduced tensile strain capacity and more brittle behavior. Similar long-term behaviors are observed from other literatures.^25,58^ Under CO_2_ curing, apart from the hydration reaction, carbonation reaction also incorporates as curing progresses. Frictional bonding between PE fibers and the matrix increases due to matrix densification from both hydration and early age carbonation. Since carbonation reacts directly with calcium hydroxide, it proceeds faster than the hydration reaction and can dominate at early age curing. From a longer-term perspective, the rapid early consumption of CH during carbonation may stimulate additional calcium trisilicate (C_3_S) hydration, but it simultaneously lowers the Ca^2+^ concentration required to sustain subsequent hydration and pozzolanic reactions. The formation of a CC̅ densified surface layer further limits CO_2_ ingress and restricts ionic transport, while the reduced availability of CH, whose generation proceeds more slowly at later ages, constrains continued matrix development. These combined effects suppress later age hydration and pozzolanic activity and help explain the close tensile responses observed in CO_2_ cured specimens at 3, 7, and 28 days.

#### 3.2.3 Crack-Pattern Characterization

The crack width distributions presented in Figure 12 provide direct microstructural evidence of the failure mechanisms governing the composite’s ductility. Generally, the crack widths followed a log-normal distribution, with the majority of microcracks concentrated in the 40–80 μm range, confirming the steady-state cracking behavior characteristic of DWC materials.

**Figure 12:**
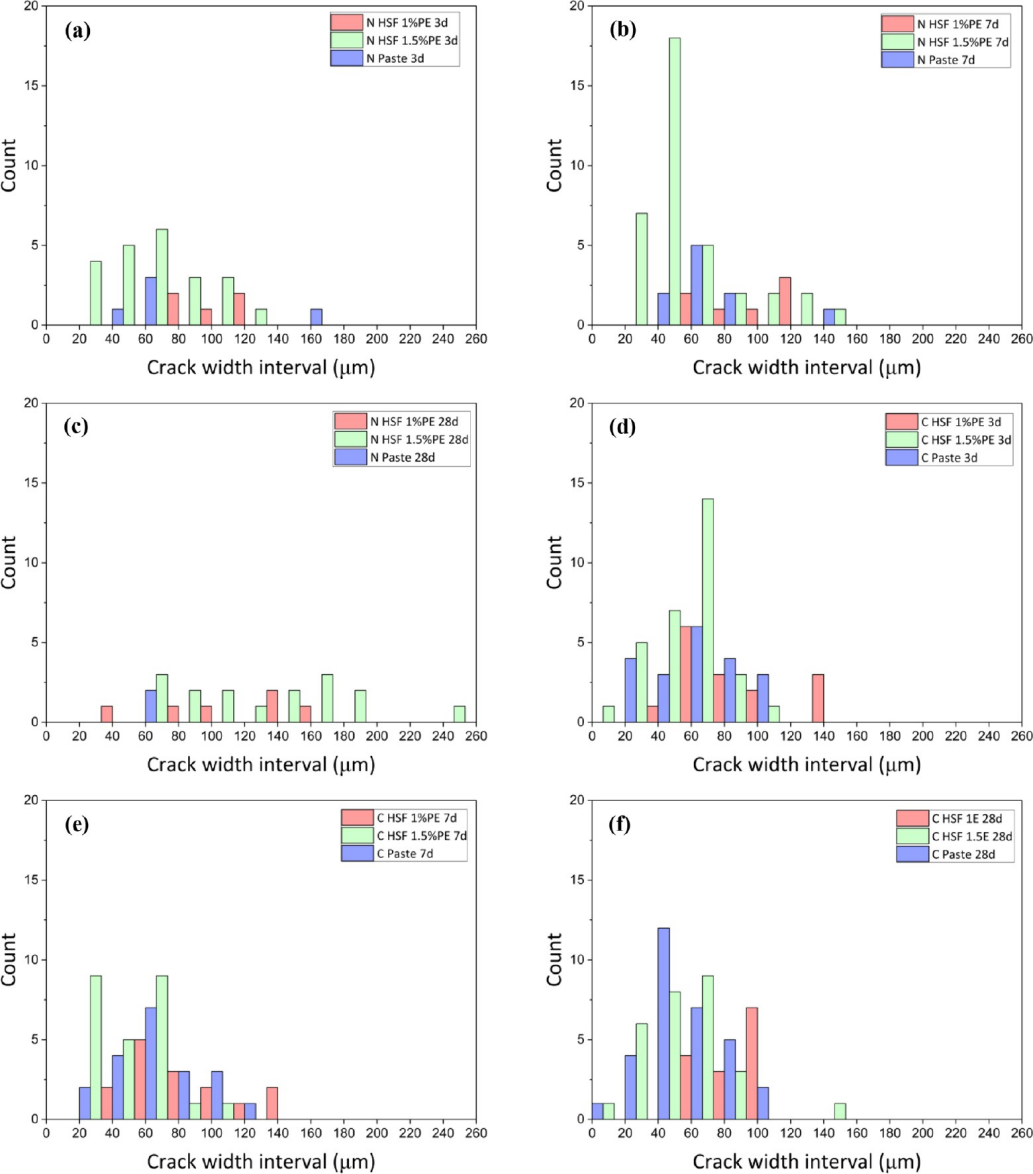
Histogram of crack width distributions for each mix design under normal (N) and CO_2_ (C) curing conditions under different ages. (a–c) Sample under normal curing conditions under 3, 7, and 28 days; (d–f) sample under CO_2_ curing conditions under 3, 7, and 28 days. One representative specimen was tested for each age and curing condition.

Under normal curing conditions, the 7-day HSF-1.5 specimen (Figure 12c) exhibited the most robust performance, displaying a sharp, high-frequency peak with over 15 cracks tightly clustered around 50 μm. This dense formation of fine microcracks correlates directly with the peak tensile strain capacity of 6.3% observed in Figure 12. However, by 28 days, the normal-cured specimens suffered significant embrittlement; the number of cracks dropped drastically, and the distribution broadened significantly to include wider cracks (140–180 μm). This coarsening suggests that as the matrix hydrated and toughened over time, the fiber-bridging capacity was exceeded, leading to crack localization rather than multiple cracking.

In contrast, CO_2_ curing effectively stabilized crack development at later ages. Although the CO_2_ cured specimens at 7 days exhibited a slightly lower crack frequency than those under normal curing, and likely due to enhanced fiber matrix bond strength restricting fiber slip, they retained a significantly finer crack structure at 28 days. Unlike the air-cured samples, the 28-day CO_2_ specimens (Figure 12f) maintained a distinct population of microcracks below 100 μm. This preservation of multiple cracking capability is the physical mechanism responsible for the sustained 4% tensile strain capacity observed in the mechanical results (Section 3.2.2). It confirms that carbonation curing prevents the late-age embrittlement of mature cementitious composites, ensuring a strain hardening response is maintained even as the matrix densifies. Beyond its mechanical advantages, the narrow crack width, particularly after CO_2_ curing, could also contribute to the durability and long-term integrity of the cement. This is particularly beneficial in aggressive well environments, as it limits crack opening and reduces pathways for corrosive fluid ingress.^59^

### 3.3 Machine Learning Results and Interpretation

As shown in Figures 13 and 14, the predictive performance of the Random Forest (RF) and XGBoost (XGB) models confirms that the selected mixture-level and microstructural descriptors adequately capture the mechanisms governing mechanical behavior. Among the three targets, compressive strength is predicted with the highest accuracy, with *R* ^2^ values of 0.95 (RF) and 0.96 (XGB), and RMSE values below 3.2 MPa. Predicted–measured plots for compressive strength show tight clustering around the 1:1 line for both models, indicating strong agreement across the full strength range. XGB exhibits marginally lower MAE and RMSE than RF, consistent with its slightly improved handling of nonlinear interactions in the data set. Prediction accuracy decreases for tensile strength and strain capacity, reflecting the inherently higher experimental variability of tensile and ductility measurements in this DWC system. For tensile strength, RF outperforms XGB with an *R* ^2^ of 0.75 compared to 0.71, and lower RMSE (0.58 vs 0.62 MPa), suggesting better robustness for this target under limited sample size. Strain capacity is the most challenging response to predict, with *R* ^2^ values of 0.62 (RF) and 0.56 (XGB). Residuals for strain capacity show larger scatter, particularly at higher measured values, indicating that sometimes brittle nature of the matrix and fiber pull-out variability introduce noise beyond what can be fully captured by global mixture and microstructural descriptors.

**Figure 13:**
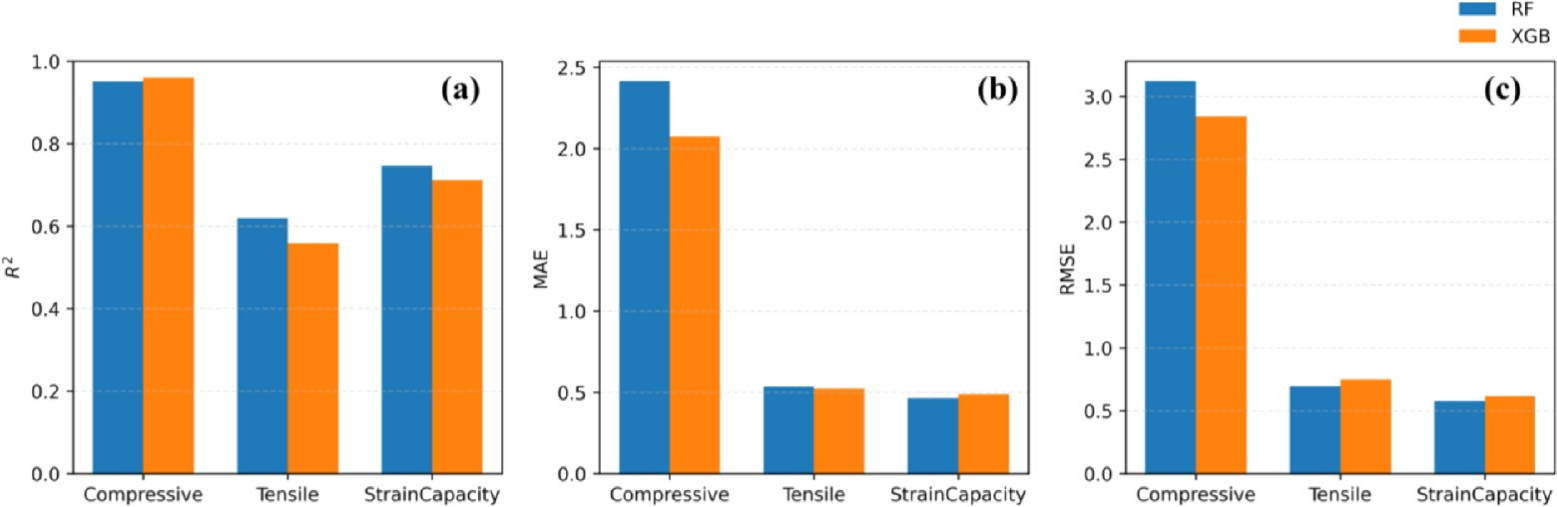
Comparison of predictive performance metrics for Random Forest (RF) and XGBoost (XGB) models in predicting mechanical properties: (a) R^2^, (b) MAE, and (c) RMSE.

**Figure 14:**
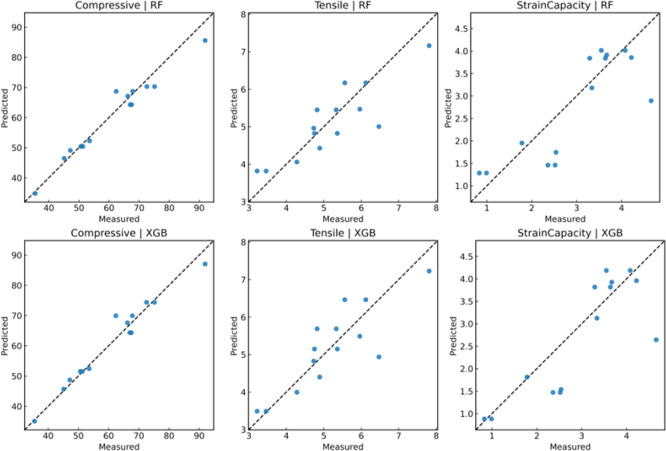
Predicted vs measured mechanical properties.

Figure 15 shows no systematic bias for either model. Errors are generally centered around zero across the measured ranges, and no consistent over- or under-prediction trends are observed. Larger residuals occur sporadically at high compressive strength (>85 MPa) and high strain capacity (>4%), reflecting edge cases where localized microstructural heterogeneity or crack localization dominates the response.

**Figure 15:**
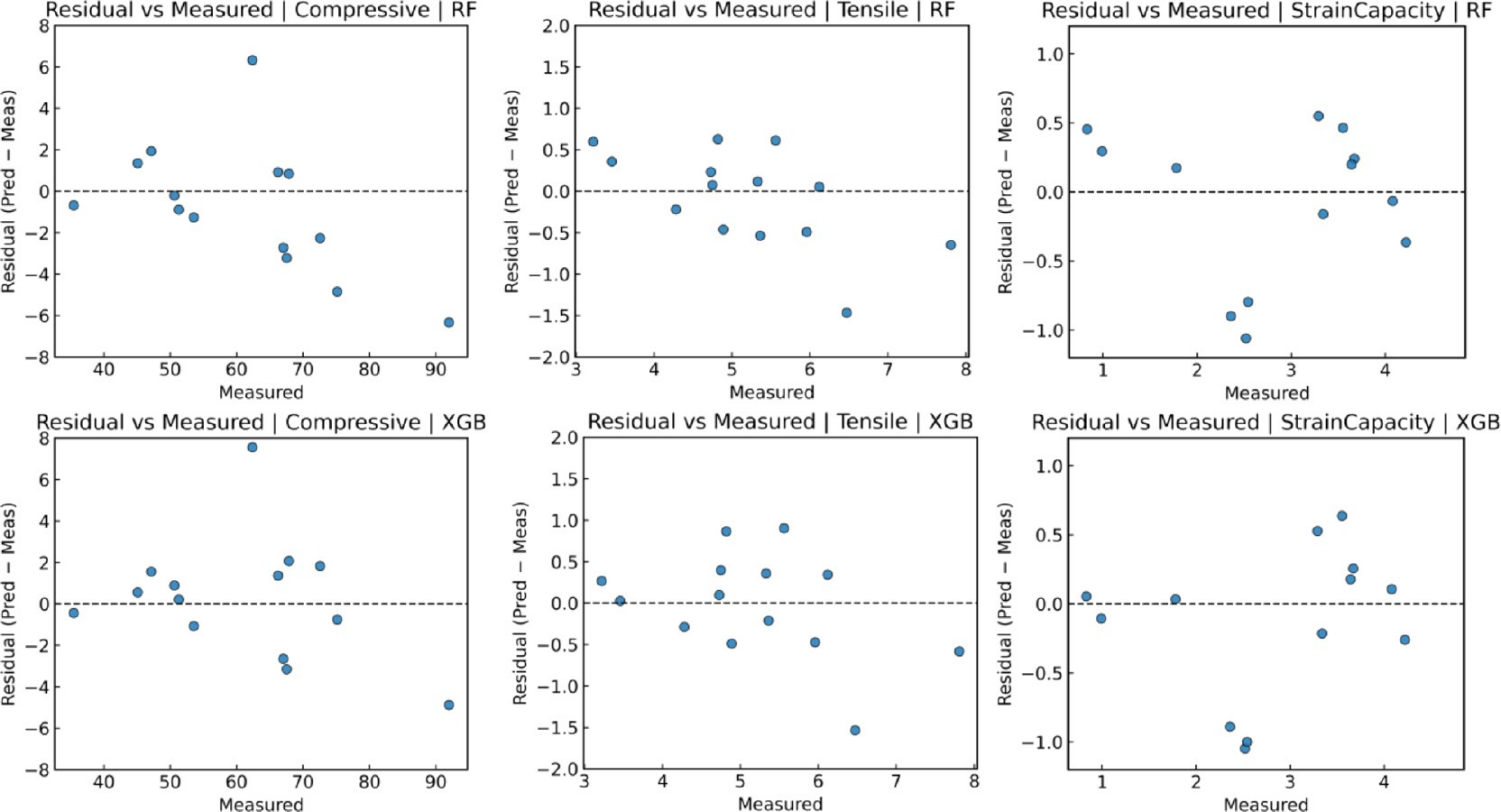
Residuals as a function of measured mechanical properties.

It can be seen from Figure 16 that feature importance rankings from RF and XGB are broadly consistent, reinforcing the robustness of the identified controlling variables. For compressive strength, microstructural descriptors associated with carbonation and hydration dominate both models. XRD CC̅ peak intensity and TGA mass loss in the 600–750 °C range consistently rank among the top predictors, highlighting the central role of carbonate formation and matrix densification. Mixture-level parameters such as binder composition and curing age appear as secondary contributors, indicating that their influence is largely mediated through microstructural evolution rather than direct mechanical control. For tensile strength, both models emphasize the combined influence of matrix chemistry and fiber reinforcement. XRD indicators of CH and CC̅, together with TGA mass-loss features, rank highly, while fiber volume fraction consistently appears among the top predictors. This reflects the dual control of tensile capacity by matrix fracture resistance and fiber–matrix interaction. Excessive matrix densification enhances peak tensile stress but may reduce the effectiveness of fiber pull-out, consistent with experimental trends observed under advanced curing and carbonation. Strain capacity exhibits a distinct importance pattern relative to strength metrics. Fiber volume fraction is the dominant variable in both RF and XGB models, confirming that ductility is primarily governed by fiber bridging and pull-out mechanisms. Nevertheless, microstructural descriptors related to CC̅ formation and CH consumption also rank prominently, indicating that matrix chemistry indirectly regulates strain capacity by modifying interfacial bond strength and crack localization behavior.

**Figure 16:**
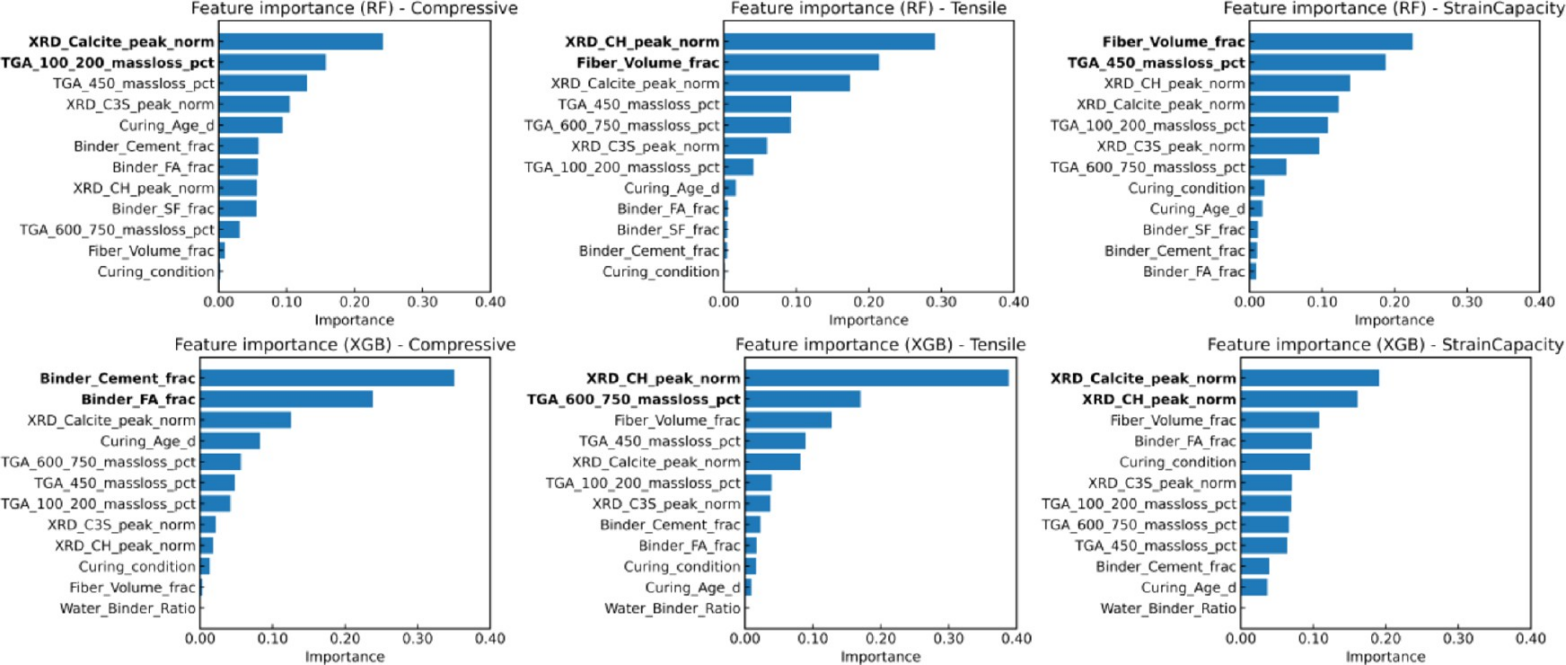
Feature importance rankings for mechanical properties.

Figure 17 presents the SHAP summary plots for the XGBoost (XGB) models. SHAP analysis was performed exclusively on XGB to provide a consistent and direction-sensitive interpretation of feature contributions within a single model framework. As SHAP values are inherently model-specific, restricting the analysis to XGB avoids potential inconsistencies across different model types. Moreover, XGB demonstrated slightly superior predictive performance for key targets, making it a robust and representative model for mechanistic interpretation. The SHAP results reveal that higher fiber volume fractions consistently contribute positively to strain capacity, confirming their role in enhancing ductility through fiber bridging. In contrast, increasing CC̅ content and degree of carbonation exhibit competing effects: moderate levels promote multiple cracking and ductility, whereas excessive matrix densification restricts fiber slip and reduces deformation capacity. Similar trade-offs are observed for tensile strength, where hydration- and carbonation-induced matrix strengthening increases peak stress but may limit tensile deformation.

**Figure 17:**
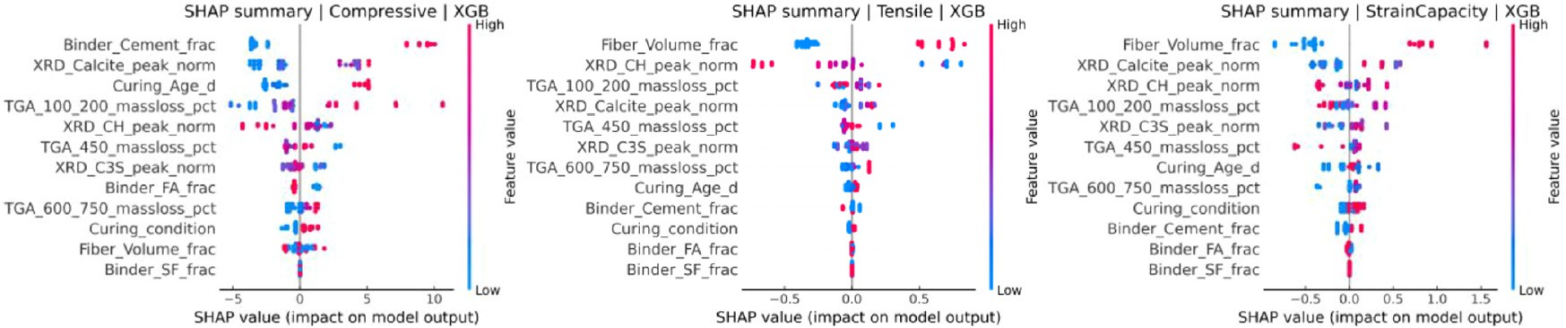
SHAP summary plot illustrating the influence of compositional and microstructural features on predictions of the XGB model.

The results corroborate the experimental observations that mechanical performance in bendable Class H cement composites is governed by a balance between fiber reinforcement and matrix evolution. Hydration and carbonation chemistry control matrix stiffness and fracture resistance, while fiber volume fraction dictates the ability to sustain multiple cracking and strain hardening. By combining predictive performance with explainable interpretation, the ML framework strengthens the causal link between microstructural development and macroscopic mechanical behavior, providing a quantitative basis for optimizing ductile well-cement formulations.

## 4 Conclusion and Future Outlook

This study examined the mechanical behavior and microstructural evolution of PE fiber-reinforced Class H cement composites containing silica flour and fly ash, with a focus on their potential application in EOR/EGR wellbores under carbonation curing. The major findings are as follows:

1. PE fibers effectively transformed the failure mode from brittle fracture to ductile strain hardening behavior, producing multiple microcracks rather than a single catastrophic break. Higher fiber content will increase the ultimate tensile stress in both curing conditions. Highest tensile ductility around 6.3% was achieved in 7 days HSF-1.5 samples. Under CO_2_ curing conditions, higher fiber content could also increase the ingress channels that allow more CO_2_ to penetrate deeper, so that the CC̅ precipitation or matrix densification can lead to higher compressive and tensile strengths. Although higher fiber contents significantly improved ductility under normal curing, CO_2_ curing introduced a trade-off: matrix densification increased ultimate tensile strength (e.g., a 14.6% gain for HSF-1.5) but reduced strain capacity (from 6.3 to 3.9% at 7 days) due to stronger fiber–matrix interfacial bonding that restricted fiber slip.
2. CO_2_ curing accelerated hydration and dramatically increased carbonate formation, as evidenced by TGA and XRD. The HSF blend achieved 11.0 wt % CO_2_ uptake at 7 days, which is five times that of pure paste, confirming its superior carbon sequestration capacity. The resulting CC̅ formation provided nucleation sites that promoted late-age hydration, explaining the fact that strength gaining get faster overtime. The tensile strain results showing a more stabilized trend this could be attributed to CO_2_ curing accelerates early strength development, but the carbonation reaction also competes with hydration by consuming CH, thereby limiting the extent of later age hydration.
3. The combination of fly ash and silica flour produced complementary benefits that depended on the curing environment. Fly ash improved workability and fiber dispersion through its spherical morphology, while silica flour effectively tuned matrix fracture toughness, enabling a peak ductility of 6.3% under 7-day normal curing. Under CO_2_ curing, however, the chemical synergy of the blend became dominant. Isothermal calorimetry showed that the slower hydration kinetics of the pozzolanic system prevented early barrier formation, allowing deeper and more complete carbonation. XRD confirmed near complete portlandite consumption and extensive CC̅ precipitation, enabling up to 17.4 wt % CO_2_ uptake by 28 days. This reaction-driven densification produced a refined, high-strength microstructure well suited for long-term zonal isolation.
4. Machine learning analysis further corroborated the experimental findings by quantitatively linking mechanical performance to mixture design and microstructural evolution. In particular, hydration- and carbonation-related descriptors dominated compressive and tensile strength predictions, while fiber volume fraction governed strain capacity, confirming that ductility arises from a balance between matrix densification and fiber pull-out mechanisms.

For future studies, this work could extend to high-temperature high-pressure (HTHP) mechanical property testing, full API tests of rheology, fluid loss and permeability, and long-term durability tests. Overall, this research fills the gap of quantifying the tensile and multicrack behavior of well cements, which are often overlooked relative to compressive strength alone. The demonstrated potential of bendable, fiber-reinforced, carbon-cured DWCs provides a promising pathway for improving the mechanical resilience of EOR/EGR wells and can enable the safe repurposing of aging wells for CO_2_ storage and geothermal energy, advancing both subsurface industrial resilience goals.

## Footnotes

1 Standard cement chemistry abbreviations are used in this paper: C = CaO; S = SiO_2_; A = Al_2_O_3_; C̅=CO_2_, and H = H_2_O.
